# The impact of transformational leadership on workers’ personal resources: latent profile analysis and links with physical and psychological health

**DOI:** 10.1192/bjo.2024.729

**Published:** 2024-08-01

**Authors:** Daniel Cortés-Denia, Manuel Pulido-Martos, Janine Bosak, Esther Lopez-Zafra

**Affiliations:** Department of Psychology, Universidad de Jaén, Jaén, Spain; DCU Business School, Dublin City University, Dublin, Ireland

**Keywords:** Physical activity, physical/psychological health, transformational leadership, vigour at work, work engagement

## Abstract

**Background:**

Several studies have examined the impact of leadership on employee well-being and health. However, this research has focused on a variable-centred approach. By contrast, the present study adopts a person-centred approach.

**Aims:**

To (a) identify latent ‘resources’ profiles among two samples combining vigour at work, work engagement and physical activity levels; (b) examine the link between the identified profiles and indicators of psychological/physical health; and (c) test whether different levels of transformational leadership determine the probability of belonging to a particular profile.

**Method:**

Two samples of workers, S1 and S2 (*N*_S1_ = 354; *N*_S2_ = 158), completed a cross-sectional survey before their annual medical examination.

**Results:**

For S1, the results of latent profile analysis yielded three profiles: spiritless, spirited and high-spirited. Both high-spirited and spirited profiles showed a positive relationship with mental health, whereas spiritless showed a negative relationship. For S2, two profiles (spirited and spiritless) were replicated, with similar effects on mental health, but none of them was related to total cholesterol. In both samples, transformational leadership determined the probability of belonging to a particular profile.

**Conclusions:**

Transformational leadership increased the probability of belonging to a more positive profile and, therefore, to better workers’ health.

Personal and job resources and motivational variables can have great effects on work-related well-being^1^ and the psychological and physical health of workers.^2^ From this point of view, the job demands–resources (JD-R) theory^3^ emerged to explain the relationship between work environment structure and work-related well-being through two main paths. The first path includes job demands, which requires sustained psychological and physical efforts for the psychological, physical, social and organisational aspects; and the second path includes job resources, such as those psychological, physical, social and organisational aspects that allow achievement of work goals, development of personal growth and reduction of the cost of job demands. Subsequently, personal resources were included; these include people's beliefs about their control over their environment^3^ and their impact on work engagement, within a profit spiral along with job resources. Several studies have investigated the implications of job resources (e.g. transformational leadership (TFL)) on personal resources (e.g. vigour at work and physical activity) and motivational variables (e.g. work engagement) and their effects on workers’ health. Nevertheless, a person-centred approach based on a combination of vigour at work, physical activity and work engagement could help to better understand the joint effects of these variables on workers’ profiles. This novel approach, which has practical implications, involves analysis and identification of profiles or subgroups based on the levels of personal resources and motivational variables shared in that subgroup. Thus, in this study, we aimed to group employees based on their shared characteristics and to analyse the connections of these characteristics to health, following the positive path of JD-R theory.

## Transformational leadership

Leaders have crucial roles in determining the work-related well-being of their ‘followers’. However, only constructive leaders can be considered a valuable resource that prevents follower burnout, because these leaders stimulate followers’ work-related well-being and motivation and contribute to goal achievement.^4^ TFL is considered to be a form of constructive leadership, as it encourages values and priorities of followers that transcend their personal interests. It has the following characteristics: high levels of charisma or idealised influence, creating a climate of trust and acting as a reference model; high inspirational motivation, generating enthusiasm for work; promoting high intellectual stimulation, fostering creativity and problem-solving abilities; and considering all members of a workplace.^5^ In fact, TFL stimulates personal growth through access to support, opportunities and adequate resources,^6^ thereby contributing to more favourable perceptions of job characteristics (fewer demands and more resources) and higher motivation levels, both of which contribute in turn to optimal job functioning through effects on job attitudes and psychological health.^7^ Similarly, other studies have demonstrated that transformational leaders can provide useful resources to followers (e.g. increased mission valence)^8^ and foster motivational outcomes such as work engagement.^9^ TFL has also been shown to be a valuable resource with respect to followers’ well-being.^1^ However, research to date has predominantly used a variable-centred approach to analyse the relationships among leadership, work engagement and personal resources. By contrast, a person-centred approach implemented through latent profile analysis (LPA) considers an interdependent system involving divergent variables, thereby enabling identification of qualitatively different subgroups within a sample. This approach allows individuals to be treated holistically through consideration of the complex interactions among variables and their combinations.

## The impact of work engagement and personal resources on workers’ health

Work engagement has been defined as a positive and persistent emotional–motivational work-related state, characterised by vigour (e.g. having high levels of energy, resilience and persistence at the workplace), dedication (e.g. having feelings of enthusiasm, challenge and pride) and absorption (e.g. having high levels of concentration and immersion in a task).^10^ Over the past two decades, scholars in this field have studied the impact of work engagement on critical employee and organisational outcomes, including the general health of workers. To date, research on the effects of work engagement on health has mainly focused on psychological health (69.3%), followed by physical health (14.1%).^2^ Specifically, work engagement has been reported to be associated with increased mental health and decreased physical and somatic symptoms,^11,12^ heart rate reactivity and systolic blood pressure,^13^ and levels of high-sensitivity C-reactive protein (hs-CRP; a biomarker related to inflammation levels).^14^

Vigour at work is a positive personal resource that results from interactions with different elements (e.g. job conditions) within the work environment^15,16^ and encompasses the following dimensions: (a) physical strength (e.g. physical abilities); (b) emotional energy (e.g. ability to express and show to others compassion, sympathy and empathy); and (c) cognitive liveliness (e.g. high mental agility and ability to generate new ideas).^15,16^ Studies have shown that vigour at work is different and distinguishable from work engagement.^15,17^ Vigour at work has also been associated with physical health and health-related behaviours,^2^ including greater protection against diabetes (17%), lower risks of mortality (26%)^18^ and of hyperlipidaemia (using a cholesterol biomarker determined enzymatically),^19^ and lower values of hs-CRP and fibrinogen (a coagulator of vascular lesions),^20^ as well as high levels of physical activity or exercise.^19,21^

Physical activity is another personal resource and is defined as the movements of the body's skeletal muscles (including walking, sports and active recreation) that require the use or expenditure of energy.^22^ Regular physical activity can contribute to prevention, treatment and/or management of various diseases (e.g. diabetes, hypertension, heart failure), as well as maintenance of a healthy body weight and improved psychological health.^22^ At work, walking can have beneficial effects on mental health.^23^ In addition, physical activity contributes to a good blood lipid profile.^24^ Moderate-intensity exercise has shown beneficial effects on very-low-density lipoprotein triglyceride, in comparison with exercise of other intensities.^25^ Nevertheless, a recent systematic review and meta-analysis highlighted the difficulty in making conclusive recommendations regarding physical activity and determining the optimal type of exercise for health, given the high diversity of types and duration.^26^ Hence, analysing different categories of physical activity, such as intensity, duration and energy expenditure, could be of great value as a means of obtaining more accurate conclusions about its impact on health. From a person-centred perspective, various job demands and resources and personal resources have been used to obtain profiles of workers. Specifically, studies have identified profiles on the basis of participants’ work engagement, burnout levels^27^ and vigour at work among different job demands and resources,^28^ whereas physical activity has mainly been used for identifying latent trajectories in workers, considering the frequency, intensity and duration of exercise,^29^ or trajectories in the variation of physical activity during working and evening hours.^30^ However, no study has yet used a combination of work engagement, vigour at work and different categories of physical activity to identify profiles using a person-centred approach and examine their impact on physical and psychological health.

Physical health can be assessed using biomarkers (e.g. total cholesterol) obtained from blood tests. For instance, studies have found that vigour at work is associated with lipids,^19^ whereas both vigour at work and work engagement are related to hs-CRP,^14,20^ and there are positive correlations between the biomarkers hs-CRP and cholesterol.^31^

This study is based on two different samples (S1 and S2) and uses a person-centred approach to: (a) identify latent ‘resources’ profiles combining several personal resources, i.e. vigour at work, work engagement and categories of physical activity (vigorous, moderate and walking); (b) examine the links between the identified profiles and indicators of psychological (S1 and S2) and physical (S2) health; and (c) test whether different TFL levels determine the probability of belonging to a particular profile (both samples).

## Method

### Procedure

Participants completed a survey before their annual work medical examination at Quirón Prevention (a company for health surveillance and control in Spain), recruited by the first author over 6 months. The inclusion criteria were that workers: (a) volunteered to participate in the study; and (b) had at least 1 month of tenure within the organisation and had an immediate manager or supervisor. In addition, one check item was included, given that inattention could affect the estimation of the profiles. Before completing the survey, each participant was informed about the study's aims and procedures and completed an informed consent form. All procedures contributing to this research comply with the ethical standards of the relevant national and institutional committees on human experimentation and with the Helsinki Declaration of 1975, as revised in 2008. All procedures involving human participants were approved by the ethics committee of the University of Jaén (ref. NOV.19/1.PROY).

### Participants

#### Sample 1

This sample comprised 432 workers. Seventy-one were excluded owing to missing more than the 5% of the responses, having failed the attention check item or being multivariate outliers. The final sample therefore consisted of 354 workers (35.3% female) from various industry sectors. The greatest participation was from the following industry sectors: education and formation (21%); economics and business administration (19.3%); industry, mechanics, electricity and electronics (10.8%); construction (9.3%); aeronautics, transport and nautical (9.1%); and agriculture, gardening and mineralogy (6.8%). On average, participants were 43.59 years old (s.d. = 10.41; range 20 to 64 years) and had a mean tenure of 12.06 years (s.d. = 11.34; range 0.08 to 48 years).

#### Sample 2

One hundred and seventy-four workers agreed to participate in the survey and to grant the research team access to the cholesterol data from their blood tests. Each worker sent his/her blood test result via email to the researchers. Twelve workers were excluded for failing the attention check measure and four for being multivariate outliers. The final sample therefore consisted of 158 workers (50.6% were female). They were on average 45.39 years old (s.d. = 10.03; range 26 to 66 years old) and had a mean tenure of 13.54 years (s.d. = 11.09; range 0.08 to 47 years). The industry sectors with the highest levels of participation were: education and formation (54.4%); economics and business administration (21.5%); industry, mechanics, electricity and electronics (4.4%); construction (3.8%); and aeronautics, transport and nautical (3.2%).

### Measures

The indicators used to obtain the profiles were categories of physical activity, levels of vigour at work and work engagement. TFL was used as a predictor, and gender, age and tenure within the organisation were controlled. Mental health was an outcome variable in both samples, whereas physical health was used only for S2.

### Demographic variables

Participants reported their gender, age and tenure within the organisation (the last two were continuous variables).

### Transformational leadership

The Spanish version^32^ of the short Multifactor Leadership Questionnaire was used to assess workers’ perceptions of their immediate supervisor's TFL style, on a five-point scale ranging from 1 = completely disagree to 5 = completely agree. The 13-item measure included four items for idealised influence (e.g. ‘he/she is able to overcome any obstacle’), three for inspirational motivation (e.g. ‘he/she develops new ways of motivating us’), three for intellectual stimulation (e.g. ‘he/she worries about our training’) and three for individualised consideration (e.g. ‘he/she gives advice to those who need it’). Cronbach's alpha was 0.97 (S1) and 0.96 (S2).

### Physical activity

The International Physical Activity Questionnaire-Short Form^33^ is a seven-item self-report measure of physical activity recall in the past 7 days (e.g. ‘During the last 7 days, on how many days did you walk for at least 10 min at a time?’). It measures the days per week and the minutes per day spent on each level of physical activity: vigorous, moderate and walking. This questionnaire allows calculation of the energy requirements for each level or condition of activity, expressed in multiples of the rate of metabolic expenditure (METs). Thus, for each worker, a value for each activity condition was obtained, with high levels of METs for walking activity being indicative of high levels of physical activity in that condition (walking) but not in the other conditions.

### Vigour at work

The Spanish version ^34^ of the Shirom–Melamed Vigor Measure was used to assess participants’ experience of vigour at work on a seven-point rating scale ranging from 1 = almost never to 7 = almost always. This 12-item measure included five items for physical strength (e.g. ‘I feel I have physical strength’), three for cognitive liveliness (e.g. ‘I feel I can think rapidly’) and four for emotional energy (e.g. ‘I feel I am capable of investing emotionally in co-workers and customers’). Cronbach's alpha was 0.92 in both samples.

### Work engagement

The original version in Spanish^10^ of the nine-item Utrecht Work Engagement Scale includes three items for each dimension: vigour (e.g. ‘When I get up in the morning, I feel like going to work’), dedication (e.g. ‘I am enthusiastic about my job’) and absorption (e.g. ‘I am immersed in my work’). The seven-point rating scale ranged from 0 = never to 6 = always. Cronbach's alpha was 0.90 for S1 and 0.93 for S2.

### Mental health

The Spanish version^35^ of the General Health Questionnaire (GHQ-12) assesses the severity of a mental problem during the past few weeks. The GHQ-12 was rated on a four-point scale from 0 = not at all to 3 = much more than usual; however, the positive items (e.g. ‘Able to enjoy day-to-day activities’) were inverted (0 = much more than usual to 3 = not at all). Thus, high values indicated worse mental health. The total score was obtained by adding all the answers, with the resulting scores ranging from 0 to 36. Cronbach's alpha was 0.83 (S1) and 0.80 (S2).

### Physical health

One week after extraction of blood samples, workers in S2 sent by email their total cholesterol value in milligrams per decilitre. This indicator was chosen on the basis of previous studies that report an association of better physical health with total values of 5 or below.^24^

### Statistical analyses

IBM SPSS Statistics version 26 was used to calculate descriptive statistics, including means, standard deviations and correlations, as well as the Cronbach's alpha values for the scales. In addition, one-way analysis of variance (ANOVA) and Scheffe's *post hoc* test were used for unequal group sample sizes to test possible differences among the indicator variables for different profiles.

MPlus 8.6 was used to identify the profiles in each sample, based on the total score for engagement and vigour at work and the METs for vigorous, moderate and walking physical activity (indicator variables for the profiles). Standardised scores of the indicators were used to facilitate interpretation, given the differences among the measurement scales (METs versus Likert-type responses). To obtain and select the best model fit and the optimal number of profiles, for both samples, the following statistics were used: log-likelihood, free parameters, Akaike information criterion (AIC), Bayesian information criterion (BIC), sample-size-adjusted BIC (SSA-BIC), entropy, smallest class, Lo–Mendell–Rubin adjusted likelihood ratio test (LMRA) and bootstrap likelihood ratio test (BLRT). Specifically, lower values on AIC, BIC and SSA-BIC were considered to indicate the best fit of the models.^36^ Entropy (with a range from 0 to 1) was also considered in the analyses; entropy values close to 1 were considered to indicate more precision in the classification of the cases in the profiles.^37^ Moreover, it is recommended that the smallest class should not be less than 5–8%. Therefore, the *P*-values for LMRA and BLRT were considered, using TECH11 and TECH14 in MPlus, with a significance threshold of *P* < 0.05. The three-step approach^38^ was used for analyses in MPlus. In particular, the classes were estimated by considering only indicator variables of the profiles in the first step, analysing the values of the fit of the model for each increase in the number of the profiles. The next step was used to obtain the most likely profile. Finally, outcome and predictor variables were analysed as the BCH auxiliary (this procedure allows a full information maximum likelihood estimation to be obtained for outcome variables) and R3STEP auxiliary (a multinomial logistic regression analysis for predictor variables).^38^ The R3STEP auxiliary allowed us to analyse the probability and odds ratio of an individual belonging to one profile over another based on the level of changes experienced in the predictor variable, whereas the BCH auxiliary enabled analysis of the implications of the profiles for outcome variables.

## Results

Table 1 shows results in the form of means, standard deviations and Pearson correlations. In both samples, the Pearson correlations mainly showed positive relationships of TFL with vigour at work and work engagement and a negative relationship of TFL with mental health (*P* < 0.001). Further, vigour at work and work engagement were positively and significantly related (*P* < 0.001), but they were negatively related to mental health (*P* < 0.001). However, the total cholesterol biomarker, which was used as an indicator of physical health, had no significant relationship with any variable in S2.

**Table 1:** Mean scores, standard deviations and Pearson correlations across samples^a^

Variable	S1 mean (s.d.)	S2 mean (s.d.)	1	2	3	4	5	6	7	8	9	10	11
TFL	3.56 (0.97)	3.62 (0.90)	–	0.17*	0.03	−0.07	0.36***	0.45***	−0.32***	−0.10	0.04	−0.16*	−0.13
Walking PA^b^	1360.25 (1141.64)	1070.23 (896.40)	−0.06	–	0.19*	0.13	0.04	0.04	−0.05	0.13	−0.00	0.05	0.20*
Moderate PA^b^	773.45 (1138.86)	592.27 (1019.70)	0.02	0.29***	–	0.24**	0.09	0.11	0.09	0.02	0.02	0.10	0.09
Vigorous PA^b^	1978.74 (2510.48)	986.58 (1672.32)	0.02	0.29***	0.36***	–	0.30***	0.17*	0.08	−0.10	−0.08	0.03	0.07
Vigour at work	5.56 (0.81)	5.38 (0.80)	0.57***	0.01	0.14*	0.09	–	0.58***	−0.44***	−0.10	−0.11	−0.12	−0.19*
Engagement	5.15 (0.89)	5.01 (0.97)	0.51***	−0.05	−0.03	0.07	0.58***	–	−0.32***	−0.03	0.02	0.04	−0.06
Mental health	13.04 (0.93)	14.23 (5.44)	−0.46***	−0.04	−0.13*	−0.10	−0.52***	−0.48***	–	0.10	0.14	0.16*	0.18*
Cholesterol (total)	–	202.44 (37.29)	–	–	–	–	–	–	–	–	−0.04	0.33***	0.33***
Gender^c^	125 (35.3)	80 (50.6)	−0.00	−0.01	−0.11*	−0.24***	−0.13*	0.01	0.14*	–	–	−0.17*	−0.13
Age, years	43.59 (10.41)	45.39 (10.03)	−0.17**	0.04	0.00	−0.06	−0.11*	−0.02	0.12*	–	0.02	–	0.75***
Tenure, years^d^	12.06 (11.34)	13.54 (11.09)	−0.16**	0.02	−0.03	0.02	−0.09	−0.01	0.16**	–	0.06	0.67***	–

TFL, transformational leadership; PA, physical activity; S1, sample 1; S2, sample 2.

a. For S1, *N*  = 354; for S2, *N* = 158.

b. The mean and s.d. values refer to the multiples of the rate of metabolic expenditure.

c. The mean and s.d. values refer to the number and percentage of female workers (male = 1, female = 2).

d. Years as a worker in the organisation. Values for S1 are in the lower part of the table; for S2, see the upper part.

*P < 0.05, **P < 0.01, ***P < 0.001.

### LPA and ANOVA to test differences among profiles

**Fig. 1:**
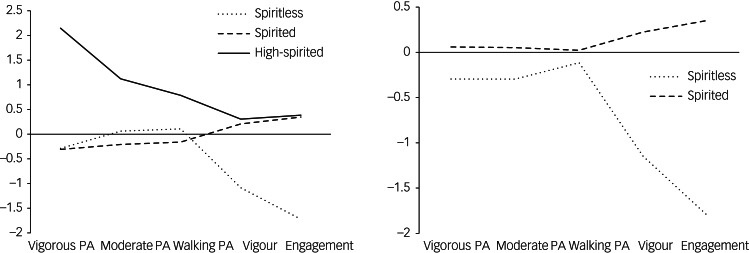
Latent profiles across samples. Left: sample 1; right: sample 2. PA, physical activity.

For S1, three profiles yielded the optimal solution (AIC = 4466.38; BIC = 4551.51; SSA-BIC = 4481.72; entropy = 0.88; smallest class = 11.6%; LMRA *P* = 0.007; BLRT *P* < 0.001) (Table 2). Figure 1 (left panel; S1) shows the main scores for all indicators in each profile. Specifically, the first profile described workers with very low levels of vigour at work (*M* = −1.086) and work engagement (*M* = −1.723) and medium levels of physical activity (moderately low levels of vigorous physical activity (*M* = −0.288) and average levels of moderate physical activity (*M* = 0.061) and walking physical activity (*M* = 0.108)). Therefore, it was labelled ‘spiritless’. This profile included 58 (16.38%) workers. The second profile included 255 (72.03%) workers with moderately high levels of vigour (*M* = 0.208) and engagement (*M* = 0.347) and medium to moderately low levels of physical activity (*M*_vigorous PA_ = −0.308, *M*_moderate PA_ = −0.209, *M*_walking PA_ = −0.161). This profile was labelled ‘spirited’. The third profile included 41 (11.58%) workers with moderately high levels of vigour (*M* = 0.305) and engagement (*M* = 0.384) and very high levels of physical activity (*M*_vigorous_ = 2.147, *M*_moderate_ = 1.122, *M*_walking_ = 0.787) and was labelled ‘high-spirited’.

**Table 2:** Latent profiles analysis model fit summary across samples

Model	Log-likelihood	FP	AIC	BIC	SSA-BIC	Entropy	Smallest class (%)	LMRA *P*-value	BLRT *P*-value
S1									
1	−2419.60	10	4859.19	4897.89	4866.16	1	354 (100)	–	–
2	−2329.72	16	4691.44	4753.35	4702.59	0.86	62 (17.5)	0.006	<0.001
3	−2211.19	22	4466.38	4551.51	4481.72	0.88	41 (11.6)	0.007	<0.001
4	−2184.76	28	4425.51	4533.85	4445.03	0.88	21 (5.9)	0.700	<0.001
5	−2143.12	34	4354.23	4485.79	4377.93	0.88	19 (5.4)	0.523	<0.001
6	−2104.89	40	4289.77	4444.54	4317.65	0.88	10 (2.8)	0.254	<0.001
7	−2069.13	46	4230.25	4408.24	4262.31	0.88	7 (2)	0.391	<0.001
8	−2046.49	52	4196.99	4398.19	4233.22	0.88	7 (2)	0.346	<0.001
S2									
1	−1084.41	10	2188.81	2219.44	2187.78	1	158 (100)	–	–
2	−1037.85	16	2107.70	2156.70	2106.05	0.90	27 (17.1)	0.013	<0.001
3	−988.11	22	2020.22	2087.60	2017.96	0.90	19 (12)	0.475	<0.001
4	−972.04	28	2000.08	2085.83	1997.19	0.91	6 (3.8)	0.189	<0.001
5	−926.54	34	1921.07	2025.20	1917.58	0.92	4 (2.5)	0.121	<0.001
6	−911.46	40	1902.92	2025.42	1898.80	0.91	4 (2.5)	<0.001	0.013
7	−898.12	46	1888.25	2029.13	1883.51	0.84	4 (2.5)	0.568	1.000
8	−877.72	52	1859.44	2018.70	1854.09	0.92	4 (2.5)	0.764	1.000

FP, free parameters; AIC, Akaike's information criterion; BIC, Bayesian information criterion; SSA-BIC, sample-size adjusted BIC; LMRA, Lo–Mendell–Rubin adjusted likelihood ratio test; BLRT, bootstrap likelihood ratio test; S1, sample 1; S2, sample 2.

For S2, two profiles yielded the optimal solution (AIC = 2107.70; BIC = 2156.70; SSA-BIC = 2106.05; entropy = 0.90; smallest class = 17.1%; LMRA *P* = 0.013; BLRT *P* < 0.001) (Table 2). The two profiles in S2 were comparable with two of the three profiles in S1 and were labelled accordingly (despite minor differences; Fig. 1, right panel: S2). The first profile included 27 (17.09%) workers with very low levels of vigour (*M* = −1.148) and work engagement (*M* = −1.800) and medium levels of physical activity. Owing to its similarity to the cluster found in S1, we also named it ‘spiritless’. The slight difference was derived from the moderately low levels of all forms of physical activity forms (*M*_vigorous PA_ = −0.295; *M*_moderate PA_ = −0.296: *M*_walking PA_ = −0.115) compared with the corresponding cluster in S1. The second profile had the largest membership, with 131 (82.91%) workers. It included individuals with moderately high levels of vigour (*M* = 0.224) and engagement (*M* = 0.352) and medium levels of physical activity (*M*_vigorous PA_ = 0.059, *M*_moderate PA_ = 0.052, *M*_walking PA_ = 0.023). For the reasons mentioned above, it was labelled ‘spirited’. No differences were found with respect to the different categories of physical activity within each profile.

In addition, given the similarities in physical activity levels within the profiles of each sample, particularly in the spiritless and spirited profiles, ANOVA was performed to determine the differences among physical activity levels. Furthermore, the rest of each profile's indicator variables were used for comparisons. The entropy was high and could be used to differentiate among the profiles in both samples (values >0.80), it allows, with certain guarantees, comparisons to be made among profiles with ANOVA. Thus, the probabilities of individuals belonging to each profile were obtained from the LPAs.

For S1, the ANOVA results showed significant differences among the three profiles with respect to the means of the following profile indicators: vigorous physical activity, *F*(2,330) = 323.685, *P* < 0.001; moderate physical activity, *F*(2,330) = 41.000, *P* < 0.001; walking physical activity, *F*(2,330) = 17.964, *P* < 0.001; vigour at work, *F*(2,351) = 53.771, *P* < 0.001; and engagement, *F*(2,351) = 313.911, *P* < 0.001). Nevertheless, Scheffe's *post hoc* test yielded no statistically significant differences between spiritless and spirited with respect to vigorous physical activity (*P* = 0.939), moderate physical activity (*P* = 0.091) or walking physical activity (*P* = 0.185), but spiritless and spirited showed differences compared with high-spirited (*P* < 0.001) in all the physical activity categories. For the remaining indicators, there were statistically significant differences in vigour and engagement for spiritless compared with spirited and high-spirited (*P* < 0.001), but not for spirited compared with high-spirited (*P* = 0.602 for vigour; *P* = 0.976 for engagement).

For S2, the ANOVA results showed no significant differences between spiritless and spirited in levels of vigorous physical activity (*F*(1,148) = 2.224, *P* = 0.138), moderate physical activity (*F* (1,148) = 1.563, *P* = 0.213) or walking physical activity (*F*(1,148) = 0.435; *P* = 0.511). Nonetheless, vigour (*F* (1,156) = 60.142, *P* < 0.001) and engagement (*F*(1,156) = 306.485, *P* < 0.001) showed significantly differences between these two profiles.

### Outcomes

Mental health differed significantly between spiritless and spirited in both samples (*P* < 0.05) (Table 3). Nevertheless, high-spirited differed significantly from spiritless but not from spirited in S1. These results show that workers who had moderately high levels of vigour and engagement and medium or high levels of all categories of physical activity (spirited and high-spirited, respectively) had better mental health than those with very low levels of vigour and engagement, and medium levels of physical activity (spiritless). However, medium or high levels of physical activity, in the spirited and high-spirited profiles, did not yield large differences in mental health. Regarding physical health, as measured by the total cholesterol biomarker, there were no statistically significant differences between spirited and spiritless in S2.

**Table 3:** Three-step results for outcome variables (BCH) across samples^a^

Profiles	S1	S2	
	Mental health	Mental health	Total cholesterol
Spiritless (A)	0.963^BC^	0.693^B^	0.004
Spirited (B)	−0.198^A^	−.135^A^	−0.001
High-spirited (C)	−0.188^A^	−	−
Chi-squared (χ^2^)	52.855***	12.640***	0.000

a. The outcome values are means. The χ^2^ value reflects the significance of the omnibus difference test. The results of pairwise comparisons are shown as superscript letters, indicating profiles that are significantly different with least at *P* < 0.05 within each row.

*** *P* < 0.001.

### Predictors

TFL affected the probability of a worker belonging to a particular profile, particularly that of whether they belonged to the spirited or high-spirited profile versus spiritless in both samples (Table 4). A one-unit increase in perception of a supervisor's TFL style quadrupled the probability of belonging to the spirited compared with the spiritless profile (odds ratio = 4.107; *P* < 0.001) and almost quadrupled the probability of belonging to the high-spirited compared with the spiritless profile (odds ratio = 3.725; *P* < 0.001) in S1; moreover, it almost tripled the probability of belonging to the spirited compared with the spiritless profile (odds ratio = 2.893; *P* < 0.001) in S2. However, TFL had no statistically significant effect on whether workers belonged to the high-spirited versus the spirited profile (odds ratio = 0.907; *P* = 0.681) in S1.

**Table 4:** Three-step results for predictor and control variables (R3STEP) across samples

		Comparison of profiles														
	Sample 1												Sample 2			
	Spirited versus spiritless				High-spirited versus spiritless				High-spirited versus spirited				Spirited versus spiritless			
Antecedent	Coef.	s.e.	OR	*P*-value	Coef.	s.e.	OR	*P*-value	Coef.	s.e.	OR	*P*-value	Coef.	s.e.	OR	*P*-value
TFL	1.413	0.222	4.107	<0.001	1.315	0.301	3.725	<0.001	−0.098	0.238	0.907	0.681	1.062	0.244	2.893	<0.001
Gender	−0.279	0.412	0.757	0.499	−1.112	0.568	0.329	0.050	−0.833	0.464	0.435	0.073	−0.117	0.562	0.890	0.835
Age, years	−0.020	0.026	0.980	0.426	−0.023	0.032	0.978	0.480	−0.002	0.021	0.998	0.918	0.102	0.048	1.107	0.036
Tenure, years	0.401	0.233	1.493	0.085	0.329	0.352	1.389	0.350	−0.072	0.292	0.930	0.805	−0.968	0.423	0.380	0.022

Coeff., coefficient; OR, odds ratio; TFL, transformational leadership.

Regarding the control variables, gender was not significant, whereas age and tenure in the organisation were only significant with respect to spirited versus spiritless in S2. In particular, a 1-year increase in age produced a 1.11-fold increase in the probability of belonging to the spirited versus the spiritless profile (odds ratio = 1.107; *P* = 0.036). However, a 1-year increase in tenure produced a four-fold increase in the probability of belonging to the spiritless profile (odds ratio = 0.380; *P* = 0.022) in the spirited versus spiritless comparison.

## Discussion

In this study, we adopted a person-centred approach with the following research aims: (a) to identify profiles of workers based on personal resources and work engagement in two samples; (b) to analyse the impact of personal resources and a motivational variable on psychological and physical health indicators; and (c) to examine whether TFL level could determine the probability of an individual belonging to the identified profiles. The model used in the present research is based on JD-R theory^3^ and considers the relationships among different personal and job resources and their effects on different aspects of health.

First, in S1, three profiles were obtained – the spiritless, spirited and high-spirited profiles. In S2, only the spiritless and spirited profiles were identified, possibly owing to the sample size. However, the two profiles obtained in S2 were similar to those found in S1. The spiritless profile reflected very low levels of vigour at work and work engagement, and medium levels of physical activity. The spirited profile showed moderately high levels of vigour at work and engagement, and medium to moderately low levels of physical activity. The high-spirited profile, in S1 only, reflected moderately high levels of vigour and engagement and very high levels of physical activity. Although Stenholm et al^30^ found large variations in daily physical activity (in amount and timing), our study indicates that workers who have high or medium levels of physical activity will probably have similar levels for the other categories. Similar results were found for vigour at work and engagement. The majority of workers were identified as belonging to the spirited profile in both samples (72.03% in S1 and 82.91% in S2), showing a moderately active lifestyle and high enough levels of vigour and work engagement levels.

We further examined whether the profiles were related to mental health. Both the high-spirited and spirited profiles were positively associated with mental health, whereas the spiritless profile was negatively associated. Thus, workers with high or medium physical activity levels and moderately high levels of vigour at work and engagement had better mental health, whereas workers with low resources were worse off in terms of their mental health.

These results are consistent with those of several studies demonstrating that various resources protect employees’ mental health;^2,12^ however, our person-centred approach also provides novel insights. Specifically, for workers who had similar levels of physical activity (e.g. the spiritless and spirited profiles), mental health was mainly determined by high or low levels of vigour at work and engagement. Moreover, workers with moderately high levels of vigour at work and engagement (e.g. high-spirited and spirited) showed a positive effect on mental health, independently of performing high or medium physical activity levels. Therefore, changing from medium to high physical activity levels did not alter the results on mental health. Thus, a medium level of physical activity should be enough to improve mental health in such individuals, and levels of physical activity exceeding certain values are not always beneficial.^23^ Concerning physical health in S2, the spiritless and spirited profiles were not related to the total cholesterol biomarker. Therefore, as both profiles had similar physical activity levels, we could conclude that vigour at work and engagement demonstrate no impact on physical health. These results contrast with the positive relationships that these resources showed in previous studies, both directly^19^ or via a proposed connection between hs-CRP and cholesterol.^31^ However, Ridker^39^ reported that cholesterol levels could not be predicted by CRP levels, because they pertain to different components of the disease. Also, the relationship between vigour at work and hyperlipidaemia could be mediated by other mechanisms, such as physiological variables and eating behaviours.^19^

Finally, the perceived level of TFL determined (and increased) the probability of belonging to a more positive profile (high-spirited/spirited versus spiritless). Specifically, employees’ perceptions of TFL were positively associated with personal resources (e.g. vigour at work), work engagement and physical activity levels. This finding is consistent with those of previous research showing that TFL can stimulate resources,^6^ contribute to good job functioning,^7^ and improve workers’ motivation and energy^9^ and highlighting the important role of leaders in the workplace.^9^ However, TFL could not be used to differentiate between the high-spirited and spirited profiles. Despite leaders having a central role in promotion of health in the workplace, their influence over workers’ physical activity is still an open question. Studies show that leaders who are healthy lead better, and that leaders who lead better are also healthier.^40^ However, when asked about their health-oriented behaviours towards employees, leaders usually refer to communication, building trust, support in boundary management and implementation of personal meetings, whereas physical activity and boundary management were particularly mentioned as health-oriented self-leadership behaviours. This could explain the lack of differentiation between high-spirited and spirited profiles with TFL; such leaders may support physical activity, but the actual level of physical activity depends on the individual worker's conception of health. This finding has practical implications; transformational leaders should promote physical activity among their workers and highlight the importance of lifestyle factors and the value of specific activities with respect to health, thereby motivating them to engage in physical activity. This would further increase the effectiveness of this leadership style.

This research had some limitations, including the use of a cross-sectional design that does not allow causal claims to be made. In fact, this design did not allow us to verify any variation in workers’ profiles (changes in levels of personal and motivational resources) that occurred with changes in whether their leader was perceived as more or less transformational over time. Therefore, future research should consider using longitudinal studies and latent transition analysis to investigate whether workers’ profiles fluctuate according to changes in their leaders. Mixed models may also be necessary to acknowledge possible leader-member variations. Although we carried out two LPA in two samples to enrich the findings of the study, the reduced number of participants with biomarkers is likely to have affected the number of profiles in S2. Moreover, workers’ data were mainly obtained through self-report measures, except for the cholesterol biomarker. Therefore, future studies should use other objective measures, e.g. accelerometers, to measure physical activity levels; they should also include other biomarkers such as high-density lipoproteins, low-density lipoproteins and triglycerides, because a moderate level of intense physical activity has been shown to be positively related to high-density lipoproteins and negatively to triglycerides but has no relationship with total cholesterol or low-density lipoproteins.^24^ In addition, other studies should include other health-related behaviours (e.g. eating behaviours), because these have a potential effect on the relationships established. This research focused on personal/motivational and social resources in the workplace for the identification of profiles. However, future studies should also consider or control variables outside the workplace with respect to their potential impact within the workplace (i.e. the impact of young children, relationship problems and elderly parents needing support) and, in turn, their impact on the profiles identified here. In addition, measures of alcohol or drug consumption should be controlled. Nonetheless, the present study contributes to the existing literature examining the impact of personal resources and motivation on workers’ health^2^ and makes a novel contribution by adopting a person-centred approach.

## Data Availability

The data that support the findings of this study are available from the corresponding author, D.C.-D., on reasonable request.

## References

[1] Roczniewska M, Smoktunowicz E, Calcagni CC, von Thiele Schwarz U, Hasson H, Richter A. Beyond the individual: a systematic review of the effects of unit-level demands and resources on employee productivity, health, and well-being. J Occup Health Psychol 2022; 27: 240–57. 34780212 10.1037/ocp0000311

[2] Cortés-Denia D, Lopez-Zafra E, Pulido-Martos M. Physical and psychological health relations to engagement and vigor at work: a PRISMA-compliant systematic review. Curr Psychol 2023; 42: 765–80.

[3] Bakker AB, Demerouti E. Job demands–resources theory: taking stock and looking forward. J Occup Health Psychol 2017; 22: 273–85. 27732008 10.1037/ocp0000056

[4] Breevaart K, Bakker AB, Hetland J, Hetland H. The influence of constructive and destructive leadership behaviors on follower burnout. In Burnout at Work: A Psychological Perspective (eds MP Leiter, AB Bakker, C Maslach): 102–21. Psychology Press, 2014.

[5] Avolio BJ, Bass BM. The Full Range Leadership Development Programs: Basic and Advanced Manuals. Bass, Avolio & Associates, 1991.

[6] Amor AM, Vázquez JPA, Faíña JA. Transformational leadership and work engagement: exploring the mediating role of structural empowerment. Eur Manag J 2020; 38: 169–78.

[7] Fernet C, Trépanier S-G, Austin S, Gagné M, Forest J. Transformational leadership and optimal functioning at work: on the mediating role of employees’ perceived job characteristics and motivation. Work Stress 2015; 29: 11–31.

[8] Bosak J, Kilroy S, Chênevert D, Flood PC. Examining the role of transformational leadership and mission valence on burnout among hospital staff. J Organ Effect 2021; 8: 208–27.

[9] Martinez IM, Salanova M, Cruz-Ortiz V. Our boss is a good boss! Cross-level effects of transformational leadership on work engagement in service jobs. J Work Organ Psychol 2020; 36: 87–94.

[10] Schaufeli WB, Salanova M, González-Romá V, Bakker AB. The measurement of engagement and burnout: a two sample confirmatory factor analytic approach. J Happiness Stud 2002; 3: 71–92.

[11] Bakhshi A, Gupta R. Personal and job related correlates of employee engagement at work. Indian J Commun Psychol 2016; 12: 312–7.

[12] Dugan AG, Barnes-Farrell JL. Working mothers’ second shift, personal resources, and self-care. Community Work Fam 2020; 23: 62–79.

[13] Black JK, Balanos GM, Whittaker AC. Resilience, work engagement and stress reactivity in a middle-aged manual worker population. Int J Psychophysiol 2017; 116: 9–15. 28238816 10.1016/j.ijpsycho.2017.02.013

[14] Eguchi H, Shimazu A, Kawakami N, Inoue A, Nakata A, Tsutsumi A. Work engagement and high-sensitivity C-reactive protein levels among Japanese workers: a 1-year prospective cohort study. Int Arch Occup Environ Health 2015; 88: 651–8. 25362516 10.1007/s00420-014-0995-9

[15] Shirom A. Feeling vigorous at work? The construct of vigor and the study of positive affect in organizations. In Research in Occupational Stress and Well Being: Vol. 3. Emotional and Physiological Processes and Positive Intervention Strategies (eds PL Perrewé, DC Ganster): 135–64. Elsevier Science, 2004.

[16] Shirom A. Vigor as a positive affect at work: conceptualizing vigor, its relations with related constructs, and its antecedents and consequences. Rev Gen Psychol 2011; 15: 50–64.

[17] Wefald AJ, Mills MJ, Smith MR, Downey RG. A comparison of three job engagement measures: examining their factorial and criterion-related validity. Appl Psychol Health Well Being 2012; 4: 67–90. 26286971 10.1111/j.1758-0854.2011.01059.x

[18] Shirom A, Toker S, Jacobson O, Balicer RD. Feeling vigorous and the risks of all-cause mortality, ischemic heart disease, and diabetes: a 20-year follow-up of healthy employees. Psychosom Med 2010; 72: 727–33. 20716713 10.1097/PSY.0b013e3181eeb643

[19] Shirom A, Toker S, Melamed S, Berliner S, Shapira I. Burnout and vigor as predictors of the incidence of hyperlipidemia among healthy employees. Appl Psychol 2013; 5: 79–98. 10.1111/j.1758-0854.2012.01071.x 23457085

[20] Shirom A, Toker S, Melamed S, Berliner S, Shapira I. Vigor, anxiety, and depressive symptoms as predictors of changes in fibrinogen and C-reactive protein. Appl Psychol 2010; 2: 251–71.

[21] Isoard-Gautheur S, Scotto-di-Luzio S, Ginoux C, Sarrazin P. The relationships between off-job physical activity and vigor at work across time: testing for reciprocity. Ment Health Phys Act 2018; 14: 47–51.

[22] World Health Organization (WHO). Physical Activity. WHO, 2020.

[23] Chekroud SR, Gueorguieva R, Zheutlin AB, Paulus M, Krumholz HM, Krystal JH, et al. Association between physical exercise and mental health in 1.2 million individuals in the USA between 2011 and 2015: a cross-sectional study. Lancet Psychiatry 2018; 5: 739–46. 30099000 10.1016/S2215-0366(18)30227-X

[24] Crichton GE, Alkerwi A. Physical activity, sedentary behavior time and lipid levels in the observation of cardiovascular risk factors in Luxembourg study. Lipids Health Dis 2015; 14: 87. 26256803 10.1186/s12944-015-0085-3 PMC4530482

[25] Slentz CA, Houmard JA, Johnson JL, Bateman LA, Tanner CJ, McCartney JS, et al. Inactivity, exercise training and detraining, and plasma lipoproteins. STRRIDE: a randomized, controlled study of exercise intensity and amount. J Appl Physiol 2007; 103: 432–42. 17395756 10.1152/japplphysiol.01314.2006

[26] Nguyen TM, Nguyen VH, Kim JH. Physical exercise and health-related quality of life in office workers: a systematic review and meta-analysis. Int J Environ Res Public Health 2021; 18: 3791. 33916437 10.3390/ijerph18073791 PMC8038562

[27] Upadyaya K, Salmela-Aro K. Social demands and resources predict job burnout and engagement profiles among Finnish employees. Anxiety Stress Coping 2020; 33: 403–15. 32223447 10.1080/10615806.2020.1746285

[28] Pulido-Martos M, Cortés-Denia D, Luque-Reca O, Lopez-Zafra E. Authentic leadership and personal and job demands/resources: a person-centered approach and links with work-related subjective well-being. Curr Psychol 2023; 42: 28994–9011.

[29] Pedersen C, Halvari H, Solstad BE, Bentzen M. Longitudinal trajectories of physical activity among employees participating in a worksite health promotion intervention: a latent class growth approach. Psychol Sport Exerc 2019; 43: 311–20.

[30] Stenholm S, Pulakka A, Leskinen T, Pentti J, Heinonen OJ, Koster A, et al. Daily physical activity patterns and their association with health-related physical fitness among aging workers-the Finnish retirement and aging study. J Gerontol Series A 2021; 76: 1242–50. 10.1093/gerona/glaa193 32766774

[31] Ghule A, Kamble TK, Talwar D, Kumar S, Acharya S, Wanjari A, et al. Association of serum high sensitivity C-reactive protein with pre-diabetes in rural population: a two-year cross-sectional study. Cureus 2021; 13: e19088. 34868746 10.7759/cureus.19088 PMC8626710

[32] Lopez-Zafra E. Un intento de validación convergente al MLQ (multifactor leadership questionnaire) de bass. Rev Psicol Soc 1998; 13: 211–6.

[33] Craig CL, Marshall AL, Sjöström M, Bauman AE, Booth ML, Ainsworth BE, et al. International physical activity questionnaire: 12-country reliability and validity. Med Sci Sports Exerc 2003; 35: 1381–95. 12900694 10.1249/01.MSS.0000078924.61453.FB

[34] Pulido-Martos M, Meléndez-Domínguez M, Lopez-Zafra E. Cultural adaptation and psychometric properties of the Shirom–Melamed vigor measure (SMVM) with workers in Spain. Eval Health Prof 2019; 42: 219–32. 29020832 10.1177/0163278717734283

[35] Sánchez-López M, Dresch V. The 12-item general health questionnaire (GHQ-12): reliability, external validity and factor structure in the Spanish population. Psicothema 2008; 20: 839–43. 18940092

[36] Morin AJS, McLarnon MJW, Litalien D. Mixture modeling for organizational behavior research. In Handbook on the Temporal Dynamics of Organizational Behavior (eds Griep Y, Hansen SD): 351–79. Edward Elgar Publishing, 2020.

[37] Celeux G, Soromenho G. An entropy criterion for assessing the number of clusters in a mixture model. J Classif 1996; 13: 195–212.

[38] Asparouhov T, Muthén B. Auxiliary variables in mixture modeling: three-step approaches using mplus. Struct Equ Mod 2014; 21: 329–41.

[39] Ridker PM. C-reactive protein: a simple test to help predict risk of heart attack and stroke. Circulation 2003; 108: e81–5. 14504253 10.1161/01.CIR.0000093381.57779.67

[40] Kaluza AJ, Boer D, Buengeler C, van Dick R. Leadership behaviour and leader self-reported well-being: a review, integration and meta-analytic examination. Work Stress 2020; 34: 34–56.

